# Emerging Proteins in CRPC: Functional Roles and Clinical Implications

**DOI:** 10.3389/fonc.2022.873876

**Published:** 2022-06-10

**Authors:** Piaoping Kong, Lingyu Zhang, Zhengliang Zhang, Kangle Feng, Yiwen Sang, Xiuzhi Duan, Chunhua Liu, Tao Sun, Zhihua Tao, Weiwei Liu

**Affiliations:** ^1^ Department of Laboratory Medicine, The Second Affiliated Hospital of Zhejiang University School of Medicine, Hangzhou, China; ^2^ Department of Blood Transfusion, The Second Affiliated Hospital of Zhejiang University School of Medicine, Hangzhou, China

**Keywords:** protein, castration-resistant prostate cancer, castration-sensitive prostate cancer, marker, therapeutic resistance

## Abstract

Prostate cancer (PCa) is the most common cancer in men in the western world, but the lack of specific and sensitive markers often leads to overtreatment of prostate cancer which eventually develops into castration-resistant prostate cancer (CRPC). Novel protein markers for diagnosis and management of CRPC will be promising. In this review, we systematically summarize and discuss the expression pattern of emerging proteins in tissue, cell lines, and serum when castration-sensitive prostate cancer (CSPC) progresses to CRPC; focus on the proteins involved in CRPC growth, invasion, metastasis, metabolism, and immune microenvironment; summarize the current understanding of the regulatory mechanisms of emerging proteins in CSPC progressed to CRPC at the molecular level; and finally summarize the clinical applications of emerging proteins as diagnostic marker, prognostic marker, predictive marker, and therapeutic marker.

## Introduction

Prostate cancer (PCa) is the most common non-cutaneous cancer in men in the western world, and is responsible for about 300,000 deaths each year (1). Androgen deprivation therapy (ADT) has been thought to be the mainstay of treatment for men with advanced symptomatic prostate cancer which is called castration-sensitive prostate cancer (CSPC). While after an initial effective response, patients often stop responding and progress to castration-resistant prostate cancer (CRPC) (2). It can be identified by a rise in prostate-specific antigen (PSA), bone scan, biopsy, and/or positron emission tomography (PET) imaging of recurrent/new metastases (3). At a conference held in March 2015, 41 experts from 17 countries and regions all agreed that the diagnosis of CRPC should meet the following two conditions: i) The serum testosterone level of the castrated is <1.7 nmol/l and ii) indicating biochemical progression. Biochemical progression is characterized by the PSA expression levels increasing twice in a row from an interval of 1 week or >3 consecutive measurements with the lowest value increasing>50% and >2 g/l, and ≥2 increases in novel lesions based on bone scanning or soft tissue lesions with the corresponding evaluation criteria of the solid tumor. At current, symptom progression is not sufficient for diagnosis of CRPC (4). Diagnosis as early as possible and precise management of CRPC are the urgent need. Proteins are the ultimate targets of most anticancer drugs (5). In this regard, investigation of protein expression and its function in promoting CRPC enable discovery of potential biomarkers for diagnosis and prognosis and therapeutic drug targets of CRPC.

Recently, with the development of high-throughput technology, a majority of proteins have been studied. Recent studies have demonstrated that proteins such as 2,4-dienoyl-CoA reductase (DECRI), alpha (1, 6) fucosyltransferase 8 (FUT8), and actinin-4 (ACTN4) are regulated in CRPC (6–8). Aberrant expression of proteins may promote CRPC cell growth, invasion, and metastasis, but inhibit cell apoptosis of the tumor and antitumor drug sensitivity. The molecular mechanisms of prostate cancer progression to CRPC are not very clear to date. In this review, we summarized the altered proteins when CSPC progressed to CRPC, and we focused on the functions, mechanisms, and clinical implementation of related proteins in CRPC.

## The Expression Pattern of Emerging Proteins in CSPC to CRPC

Large amounts of proteins have been found to express differently compared CSPC with CRPC. The altered proteins in the transition from CSPC to CRPC and the relevant references are summarized in **Table 1**.

**Table 1:** The altered proteins when CSPC progression to CRPC.

Protein	Matrix	Alteration in CRPC	Proteomic technologies	Function	Clinical association	References
**PARP-2**	**Tissue**	**Increased**	**IHC**	**Promotes AR-positive CRPC growth**		(9)
**SFK**	**Tissue**	**Increased**	**IHC**	**Promotes CRPC proliferation and metastasis**	**Prognostic marker**	(10)
**SEMA3C**	**Tissue**	**Increased**	**IHC**	**Promotes CRPC growth**	**Therapeutic target**	(11, 12)
**RNF6**	**Tissue**	**Increased**	**IHC**	**Promotes CRPC growth**		(13)
**AR-V7**	**Tissue**	**Increased**	**IHC**	**Promotes CRPC growth and metastasis**	**Prognostic/predictive/diagnostic marker, therapeutic target**	(14–18)
**YAP1**	**Tissue**	**Increased**	**IHC**	**Promotes CRPC growth, invasion and FFA production**	**Therapeutic target**	(19, 20)
**PAK2**	**Tissue**	**Increased**	**IHC**	**Promotes CRPC growth and invasion**	**Therapeutic target**	(19)
**ERG**	**Tissue**	**Decreased**	**IHC**		**Therapeutic target**	(21, 22)
**ACTN4**	**Cell lines**	**Increased**	**WB**	**Promotes CRPC growth and invasion**	**Therapeutic target**	(8, 23)
**TBLR1**	**Cell lines**	**Decreased**	**WB**		**Therapeutic target**	(24)
**MDR1**	**Cell lines**		**WB**		**Therapeutic target**	(25, 26)
**p66Shc**	**Cell lines**	**Increased**	**WB**	**Promotes CRPC growth and metastasis**		(27, 28)
**VCL**	**Cell lines**	**Increased**	**LC-MS/MS**		**Therapeutic target**	(29, 30)
**FLNC**	**Cell lines**	**Increased**			**Therapeutic target**	
**EpCAM**	**Cell lines**	**Decreased**	**LC-MS/MS, WB**	**Promotes CRPC growth and invasion**	**Therapeutic target**	(31, 32)
**caspase 3**	**Cell lines**	**Decreased**			**Therapeutic target**	
**vimentin**	**Cell lines**	**Increased**			**Therapeutic target**	
**catalase**	**Cell lines**	**Increased**			**Therapeutic target**	
**BDH1**	**Cell lines**	**Increased**	**MS**		**Prognostic/diagnostic marker**	(33, 34)
**HMGCL**	**Cell lines**	**Increased**			**Prognostic/diagnostic marker**	
**HMGCS2**	**Cell lines**	**Increased**			**Prognostic/diagnostic marker**	
**ACAT1**	**Cell lines**	**Increased**			**Prognostic/diagnostic marker**	
**OXCT1**	**Cell lines**	**Increased**			**Prognostic/diagnostic marker**	
**FUT8**	**Cell lines**	**Increased**	**LC-MS/MS**	**Promotes CRPC growth and metastasis**		(7, 35, 36)
**Dicer**	**Cell lines**	**Increased**	**LC-MS/MS**			(37)
**ASPH**	**Cell lines**	**Increased**	**WB, IHC, MS**	**Promotes CRPC growth and invasion**	**Prognostic marker**	(38, 39)
**hnRNP U**	**Cell lines**	**Increased**				
**RKIP**	**Cell lines**	**Decreased**				
**DECR1**	**Cell lines**	**Increased**	**MS**	**Promotes CRPC growth and inhibits lipid metabolism**	**Prognostic marker**	(6)
**TIMP-1**	**Serum**	**Increased**	**ELISA**			(40)
**IL-4**	**Serum**	**Increased**	**ELISA**			(41)
**IL-6**	**Serum**	**Increased**				
**IL-10**	**Serum**	**Increased**				

IHC, immunohistochemistry; CRPC, castration-resistant prostate cancer; AR, androgen receptor; FFA, free fatty acids; WB, western blotting; LC, Liqui Chromatography; MS, Mass Spectrometer; ELISA, enzyme-linked immunosorbent assay.

### The Expression Pattern of Emerging Proteins in CRPC Tissue

Prostate cancer tissue could readily be obtained by prostatectomy or biopsy (42). In addition, more effective markers for CRPC diagnosis, prognosis, therapeutic management, and the mechanisms of transformation of CSPC to CRPC could be found by investigating the expression pattern of proteins in prostate cancer tissue.

Developmental changes in enzyme levels have been discovered during prostate cancer progression to CRPC. PARP-2 is a member of Poly (ADPribose) Polymerase (PARP) family which plays a critical role in AR-mediated transcription in PCa and prostate cancer progression to CRPC compared with other members (9). To explore the oncogenic role of PARP-2 in PCa, the study used a set of PCa tissue microarrays (TMAs) containing 1129 tissue cores and found that significantly increased PARP-2 protein expression was observed in CRPC compared with primary PCa tumors (9). Selective targeting of PARP-2 may provide an alternative therapeutic strategy for AR inhibition that is comparable with enzalutamide treatment through disrupting FOXA1 binding rather than targeting AR directly (9). Compared to currently used PARP in targeting both PARP-1 and PARP-2, PCa patients may benefit from selective targeting of PARP-2 because pan-inhibitors generally have more side effects than selective inhibitors (9). Although the use of Src family kinases (SFKs) inhibitors in prostate cancer cell lines has also been the subject of several publications, it is still not entirely clear which SFK member plays a dominant role in the transition to hormone independence in prostate cancer compared with PARP (10). To investigate the association between SFK activity and clinical data in prostate cancer patients, Oleg Tatarov et al. compared prostate tumor samples taken before hormone deprivation therapy and following hormone relapse from 50 patients by immunohistochemistry (10). They found that the expression of total Src and phospho-SrcY527 representing an inactive form of SFKs in the cytoplasm decreased in the transition from CSPC to CRPC, while more intense membrane staining of overall SFK member Lyn was observed in CRPC samples when compared with CSPC (10). There was no change found in the expression of SFK member Fgr (10). In addition, SFK activity was upregulated in 28% of patients with CRPC compared to patients with CSPC (10). However, only a subset of AIPC patients may be suitable for SFK inhibitor therapy. The single-agent activity of SFK inhibitors in PCa clinical trials is disappointing (11). A study has shown that Semaphorin 3C (SEMA3C) drives the activation of multiple receptor tyrosine kinase pathways including EGFR, HER2/ErbB2, MET, and SRC, so inhibition of only one pathway may not be sufficient because other compensatory receptor tyrosine kinase pathways are simultaneously activated (11). James W Peacock et al. examined the expression of SEMA3C in a panel of human PCa specimens representing untreated hormone naive and CRPC bone metastases and observed that increased SEMA3C expression was correlated with CRPC bone metastases (11). Ring finger protein 6 (RNF6), an ubiquitin E3 ligase, may potentially be a new therapeutic target of CRPC (13). Kexin Xu et al. found that nuclear staining of RNF6 in CRPC tissues was remarkably higher than those in CSPC tissues (13). Furthermore, the frequency of detection of RNF6 positive nuclear staining was significantly increased in CRPC tissues compared to the CSPC tissues (13).

In addition to kinases above, there are other proteins that change during prostate cancer progression to CRPC. A previous study discovered that yes-associated protein 1 (YAP1), which plays a crucial role in the mammalian hippo signaling pathway and AR, formed a protein complex in the nucleus of prostate cancer cells (43). In addition, the YAP1–AR interactions are androgen-independent and resistant to enzalutamide in CRPC (43). AR-V7 is truncated after canonical AR exon 3, with inclusion of a cryptic exon 3b (CE3b) derived from an intron in the expressed protein (44). Jonathan Welti et al. found that nuclear AR-V7 expression was significantly higher in CRPC compared to CSPC (14). The authors further demonstrated a reverse trend with a decrease in nuclear AR-V7 in a small subset of CRPC, which might be explained by an increase in other AR variants that are constitutively active (14). Similarly, Arnaud Blomme et al. discovered that expression of the AR-V7 variant significantly increased in Bicalutamide-resistant cells while decreased or did not change in the apalutamide- and enzalutamide resistant cells (6). Yoshinori Matsuda et al. also found that higher nuclear YAP (nYAP) expression was expressed in a docetaxel-resistant subline (22RV-1-DR) compared to parental 22Rv-1 and knockdown of YAP1 inhibited 22Rv1-DR cell proliferation (45). Another study using immunohistochemistry also demonstrated that YAP1 and p21-activated kinase 2 (PAK2), a regulator of cell motility that mediates the actions of Cdc42 and Rac small GTPases, were increased in castration-resistant tumors (19, 46, 47). Moreover, nYAP expression is an independent prognostic factor in high-risk patients treated with docetaxel-based chemohormonal therapy (45). Therefore, the interaction between AR, including its splice variants and hippo pathway proteins as tissue biomarkers in CRPC, should be further investigated. Transmembrane serine protease 2 (TMPRSS2) is a prostate-specific and androgen-responsive serine protease which is frequently rearranged at the genomic level with members of the E26 transformation-specific (ETS) gene family (48). The most frequent fusion involves the “ETS-related gene” or “ERG” occurring in approximately 40%–50% of primary PCa (21). Nuclear ERG protein expression was observed to be less frequent in CRPC compared with primary PCa by immunohistochemistry (21). HLA-DMB, a protein involved in inflammation, association with ERG was decreased in CRPC metastases compared with primary PCa (21). And CD3 cell number association with ERG was found to change from positive to negative in CRPC metastases when compared with primary PCa (21). Furthermore, expression of DCLK1 shows correlation with ERG expression and may play a role in the transformation of primary PCa to metastatic CPRC which deserves further study (21).

Several studies have found that changes in phosphorylation pattern of proteins could also be observed during transformation of CSPC to CRPC. Phosphorylation is a reversible posttranslational modification that can inform about the activity status of kinase-driven signaling pathways (19). A study found that nearly 50% of CRPC tissues showed higher expression levels of overall tyrosine phosphorylation than hormone naive prostate cancer (49). Justin M et al. analyzed metastatic CRPC and treatment naive tissues by quantitative label free mass spectrometry, and 297 phosphotyrosine peptides and 8051 hosphoserine/phosphothreonine peptides were identified from 54 total runs corresponding to 27 samples of interest (11 treatment-naive,16 metastatic CRPC) (50).

### The Expression Pattern of Emerging Proteins in Cell Lines

Cell lines are a good model for studies about prostate cancer to find the expression pattern of proteins, for the reason that different conditions and variations in patients can be mimicked to be observed and it would not have been able to be assessed otherwise. In addition, the majority of interventional studies can be carried out without being affected by other variables (42). Some proteins were discovered by traditional techniques such as western blotting (WB), while some new proteins were discovered by mass spectrometry.

Some proteins have been found expressed differently between CSPC and CRPC by traditional techniques such as WB. ACTN4, a member of the spectrin gene superfamily, always acts as an oncogene in various cancer types (23). To identify novel therapeutic targets for CRPC, Yu Ishizuya et al. applied WB to examine the expression of ACTN4 in four prostate cancer cell lines (8). It was shown that ACTN4 expression level was higher in DU145 cells and PC-3 cells than in LNCaP cells (8). Sungyeon Park et al. also verified that the protein level of ACTN4 was increased in LNCaP-AI cells compared with LNCaP cells (23). A previous study reported that ACTN4 interacts with glucocorticoid receptor and plays a role in glucocorticoid receptor activation (51). However, restored glucocorticoid receptor expression is crucial for CRPC cell proliferation during the CRPC transition (52). Therefore, the molecular mechanism of ACTN4 in CRPC transition deserves further study. Another study using WB revealed a significant deregulation of transducin beta like related 1 (TBLR1), which is a transcriptional coactivator of androgen receptor (AR) when prostate cancer progresses to CRPC (24). TBLR1, also known as TBL1XR1, a core component of the nuclear receptor corepressor complex and a silencing mediator of retinoic acid and thyroid receptor complex, which was significantly important for the regulation of multiple nuclear receptors, functions as a tumor suppressor when expressed in the nucleus in prostate (53–56). The expression level of cytoplasmic TBLR1 was moderately higher in PCa cells than benign prostate cells (24). CRPC cells express higher levels of TBLR1 cytoplasmic expression and lower levels of nuclear expression compared with CSPC (24). In addition, a cytoplasmic specific isoform of TBLR1 approximately 5 kDa was observed lower in molecular weight, which expressed higher levels in CRPC cells (24). Interestingly, Madeleine Saupe et al. discovered that one of the major contributors to chemoresistance in solid cancer entities, multidrug resistance 1 (MDR1), is of less importance for drug resistance in PCa cells (25). They examined expression of MDR1 in the established PCa cell lines 22Rv1, LNCaP, and PC-3 by WB and found that MDR1 basal expression only could be detected in the PCa cell line 22Rv1 (25). However, MDR1 expression level did not change when incubation of 22Rv1 cells with cabazitaxel, docetaxel, and abiraterone (25).

Enzymes involved in the androgen and metabolic pathway are always regulated in CRPC cells. To identify novel therapeutic targets for CRPC therapy, a study investigated the expression of p66Shc in PCa (27). p66Shc, a 66 kDa proto-oncogene Src homologous-collagen homologue adaptor protein, mediates receptor tyrosine kinase signal transduction and was identified as a sensor for apoptosis induced by oxidative stress (57). Matthew A. Ingersoll et al. discovered that LNCaP-AS cells expressed relatively lower levels of p66Shc protein compared to AR-negative AI PC-3 and DU145 cells (27). Another study also verified that p66Shc protein level was increased in LNCaP-AI and VCaP-AI cells compared with their AS counterparts (28). An interesting study demonstrated that intrinsically disordered proteins conformational dynamics also played a role in driving phenotypic heterogeneity (58). Prostate-Associated Gene 4 (PAGE4) is an intrinsically disordered protein that functions as a potentiator of the Activator Protein-1 (AP-1) transcription factor (58). Homeodomain-Interacting Protein Kinase 1 (HIPK1) acts on PAGE4 phosphorylates it at T51 and S9, while CDC-Like Kinase 2 (CLK2) hyperphosphorylates PAGE4 at multiple S/T residues, including T51 and S9 (58). HIPK1 was identified to express in both CSPC and CRPC cells, but the expression of CLK2 and PAGE4 was only discovered in CSPC cells (58). HIPK1-phosphorylated PAGE4 exhibited a relatively compact set of conformations bound to AP-1, but CLK2-phosphorylated PAGE4 was more extended, resembling random coils with reduced affinity for AP-1 (58). The result indicated that conformational dynamics of PAGE4 played a role in PCa progression to CRPC (58).

In order to identify new biomarkers, proteomic technologies such as 2DE-MS, MALDI-MS and SELDI-MS, and i-TRAQ are widely used in PCa and a majority of proteins regulated are discovered during prostate cancer progression to CRPC (59). In a study using quantitative proteomics, 203 differential proteins in total were differentially expressed in LNCaP and PC3 cells (29). Vinculin (VCL), an actin filament-binding protein, and Filamin C (FLNC), an important component of cytoskeleton, were observed to have significantly higher expression in PC3 cells when compared to LNCaP cells (29, 30).

In another study using established androgen-dependent (AD) and androgen-independent (AI) murine PCa cell lines, PLum-AD and PLum-AI, respectively, a total of 683 proteins were identified, among which 99 were significantly regulated in PLum-AI cells compared to PLum-AD cells (45 increased and 54 decreased) by Liqui Chromatography (LC)–Mass Spectrometer (MS)/MS Analysis (31). Among them, eight proteins (Comt, Rps11, Nos2, Oxr1, Pck2, Grb10, Cat, Nqo1) were only identified in PLum-AI cells while 12 proteins (Acat2, Fdps, Epcam, Casp3, Tpd52, Ap1s1, Atp6v1g1, Hnrnpul2, Xpnpep1, Hmgcs1, Oasl1, Ifit1) were exclusively observed in PLum-AD cells (31). Furthermore, EpCAM and caspase 3 were significantly decreased in PLum-AI cells, while vimentin and catalase were found to highly increase in PLum-AI cells (31). Biological process gene ontology (GO) analysis of the differentially expressed proteins demonstrated enrichment of biological functions and pathways in PLum-AI cells that are central to PI3 kinase and androgen receptor pathways (31). Besides, other relevant biological processes that are enriched in PLum-AI cells included cell adhesion and cell migration processes, cell and DNA damage, apoptosis, and cell cycle regulation (31).

To understand the mechanisms leading to CRPC transformation, the proteomes of LNCaP cell line and its androgen-independent derivative, LNCaP-SF, were compared by MS (33). The result showed that 42 proteins were upregulated while 46 proteins were downregulated in LNCaP-SF cells compared to LNCaP cells (33). Interestingly, five proteins of the ketogenesis pathway (BDH1, HMGCL, HMGCS2, ACAT1, and OXCT1) were increased in LNCaP-SF cells (33). Additionally, it was discovered that ACAT1, HMGCS2, BDH1, and HMGCL all displayed significantly higher expression in the LuCaP 96AI xenograft-derived cells (33).

Naseruddin Höti et al. found that a glycosylation-related enzyme, FUT8, was increased in CRPC and responsible for resistance to androgen deprivation by LC-MS/MS (7, 35). To dissect the mechanism of castration resistance, proteomic studies have also been conducted by comparing castration-resistant LNCaP-95 cells and LNCaP cells (37). A total of 1883 proteins altered between the two cell types (37). Significant involvement of metabolic pathways was shown to increase in LNCaP-95 cells (37). Furthermore, amplification of PI3K/AKT pathway and proteasome proteins overexpression were also discovered (37). Conversely, the mitochondrial oxidation phosphorylation was severely inhibited in castration-resistant LNCaP-95 cells relative to LNCaP cells (37). Interestingly, the author discovered that Dicer, a cytoplasmic endoribonuclease microRNA regulator, was induced in LNCaP-95 prostate cancer cells (37). Another study performed an integrative proteomic analysis and found that 15 proteins could promote androgen-resistance acquisition (38). It was also verified that 11 out of these 15 proteins correlated with biological processes involved in PCa progression (38). Comparing CSPC with CRPC, ZHANG Xiaobo et al. found that 11 membrane proteins were differently expressed, such as the aER60 precursor, Neural-Cadherin precursor, Claudin-4, and so on (60). The expression level of Claudin-4 in PC-3 cells was discovered to be higher than in LNCaP cells by Immunofluorescence (60).

Recently, a study found that 13 proteins were differentially expressed comparing AR inhibitors (ARI)-resistant cell lines with WT LNCaP cells, such as SQRDL, EPHX1 (6). Among these proteins, DECR1 was found to consistently increase in ARI-resistant cells as compared to WT LNCaP cells (6). Moreover, other proteins differentially expressed in CRPC and CSPC, such as PGP 9.5, stathmin, ICAM-1, purine-rich element binding protein α and ChA, were also discovered by proteomic studies (61–64).

In addition to changes in the expression levels, epigenetic changes have also been studied in cell lines in recent years. For instance, Ravi Pathak et al. found that the number of cellular proteins undergoing acetylation was more in the CSPC with respect to the CRPC (65).

### The Expression Pattern of Emerging Proteins in Serum

Although biopsies are considered to be the “gold standard” for the diagnosis of prostate cancer, biopsies are invasive and are associated with complications such as bleeding and sepsis. Biopsy also has the problem of false negative caused by ineffective sampling, which means it is necessary to carry out more research on non-invasive body fluids. However, in recent years, there has been little research in the area about altered proteins in serum in prostate cancer when progressing to CRPC.

Although serum PSA is widely used in the diagnosis and treatment of prostate cancer, it is highly expressed in both benign and malignant prostate epithelium. Therefore, a more specific serum marker needs to be found. Human β-2-microglobulin (B2M) was more specific for androgen stimulation under the conditions tested compared with PSA and discovered in the serum of mice which bear human prostate cancer xenograft (66). It was also found to express in human prostate cancer tissues and cell lines (66). Importantly, serum B2M levels were increased in metastatic CRPC patients (66). Further studies are warranted to investigate if B2M could be a useful marker for PCa. Another study also investigated the marker for treatment of metastatic CRPC patients by analyzing the expression levels of three EMT-related proteins (67). Integrin α4 is a ‘‘matricellular’’ protein receptor that forms a complex with integrin β7 or β1, which then adheres to fibronectin (67). Fibronectin is a crucial ECM glycoprotein as an actor in both pathological and physiological processes (67). When comparing blood samples of benign prostatic hyperplasia patients and a healthy control group with the metastatic castration-resistant prostate cancer patients, Ece Konac et al. discovered a decreased integrin-α4 expression in metastatic castration-resistant prostate cancer patients, although being statistically insignificant (67). It was significant that protein expressions of periostin and fibronectin increased in the metastatic castration-resistant prostate cancer patients compared with BPH and heathy groups (67). Elevated periostin expression in metastatic patients was identified to correlate with bone metastasis (67). Interestingly, a study demonstrated that increased serum tissue inhibitor of metalloproteinase 1 (TIMP-1) expression in CRPC correlated with neuroendocrine differentiation of PCa (40). Yixuan Gong et al. found that serum TIMP-1 expression is increased in CRPC patients compared to CSPC patients (40). Also, elevated TIMP-1 levels correlated with higher chromogranin A and lower PSA levels in sera, features consistent with neuroendocrine prostate cancer (40).

To make it clear whether the quantity of antibodies or the types of proteins changes with prostate cancer progression, serum samples from newly diagnosed localized prostate cancer, castration-sensitive non-metastatic prostate cancer, castration-resistant non-metastatic prostate cancer, and castration-resistant metastatic disease were analyzed (68). The study revealed the largest change in composition of recognized antibody targets occurred between patients with castration-sensitive and castration-resistant disease, such as C12orf51, RPL22L1, and TBL1XR1 (68). GO analysis identified differences between patients with castration-sensitive and castration-resistant disease in recognition of proteins associated with amide metabolism, chromatin structure, nucleic acid binding, and protein localization to the membrane (68). Another study using two-dimensional differential in-gel electrophoresis and matrix-assisted laser desorption/ionization time-of-flight mass spectrometry, nine proteins were found to express differently in the serum samples collected at diagnosis compared with those after ADT (69). It was further found that clusterin (CLU) expression was 1.51-fold higher and transthyretin expression was 1.58-fold lower in the sera of post-ADT patients with respect to those from pre-ADT patients (69).

It was suggested that inflammation can promote the development of prostate cancer. Evidence from lots of studies showed that many biomarkers associated with prostatic inflammation diseases also existed in prostate cancer (70). Gilbert J. Wise et al. discovered that the anti-inflammatory Th2 cytokines IL-4, IL-6, and IL-10 were significantly increased in the hormone refractory group compared with hormone sensitive group by enzyme-linked immunosorbent assay (41).

Docetaxel chemotherapy improves survival and symptoms in men with metastatic CRPC. However, 50% of patients were resistant to Docetaxel (71). Using Docetaxel-sensitive PC3 cells and Docetaxel resistant PC3-Rx cells, Liangli Zhao et al. found that MIC-1 and AGR2 were increased and decreased in Docetaxel resistant cells by protein profiling in serum (71). Caspase-cleaved proteins are released from disintegrated apoptotic cells, which could be detected in the circulation (72). G Kramer et al. evaluated a total of 82 patients with CRPC and discovered that caspase-cleaved cytokeratin 18 (CK18-Asp396) and total CK18 were significantly upregulated induced by Docetaxel in serum (72).

## The Function of Emerging Proteins in CRPC

**Figure 1:**
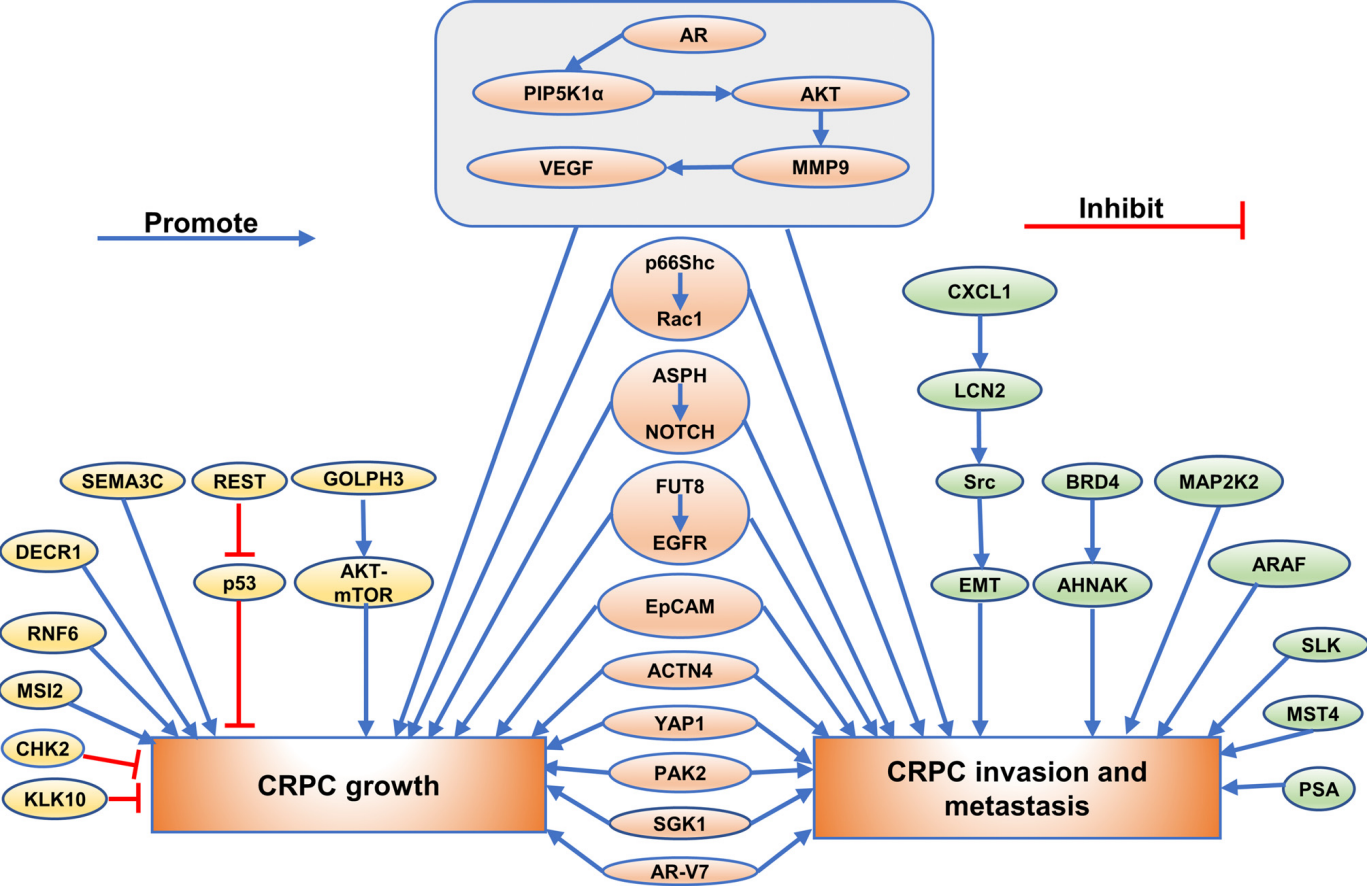
The role of emerging proteins in CRPC growth, invasion and metastasis. The emerging proteins involved in CRPC growth are showing as golden, and the proteins involved in CRPC invasion and metastasis are showing as green. The proteins involved in both CRPC growth and CRPC invasion and metastasis are showing as orange.

**Figure 2:**
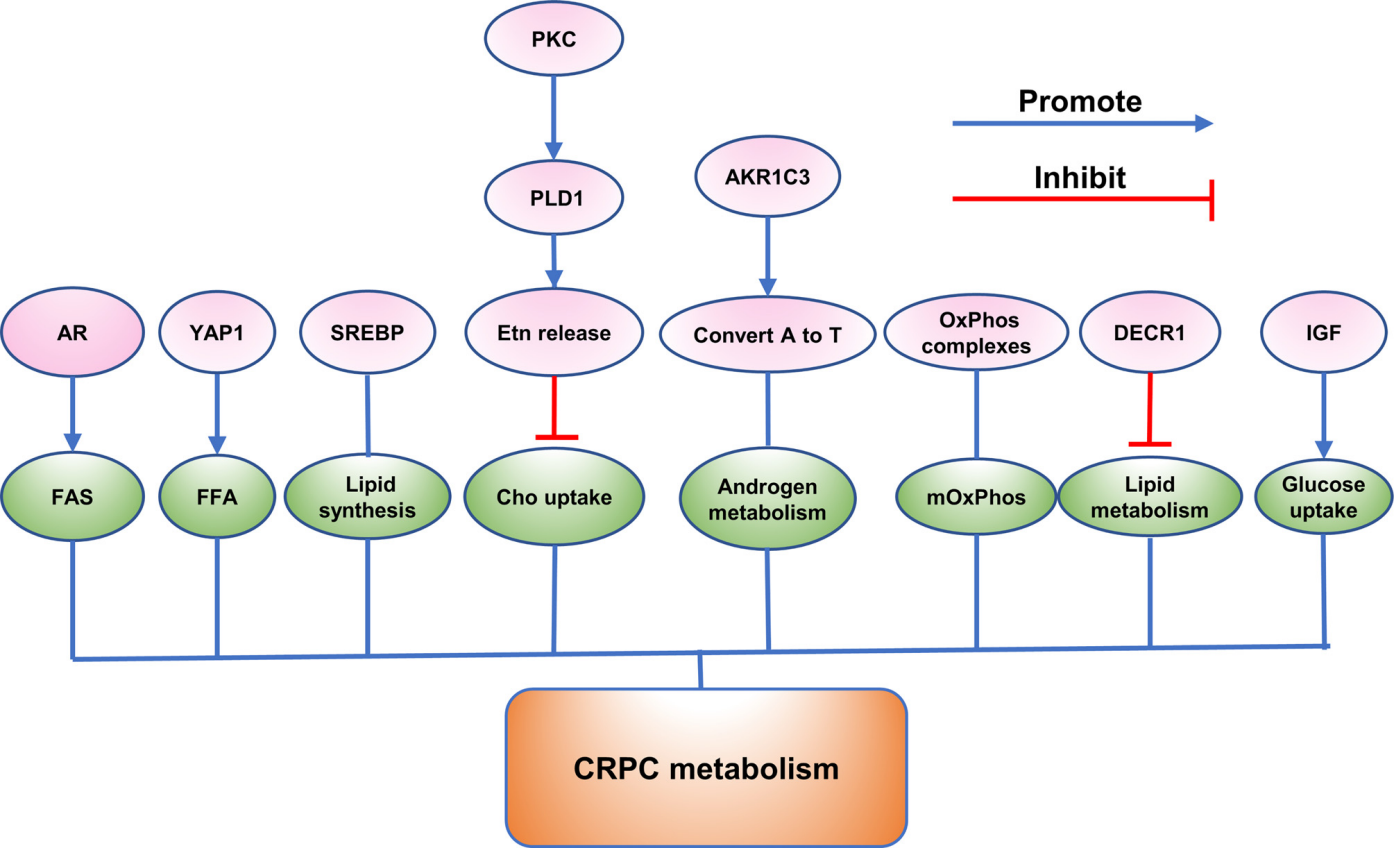
The role of emerging proteins in CRPC metabolism.

**Figure 3:**
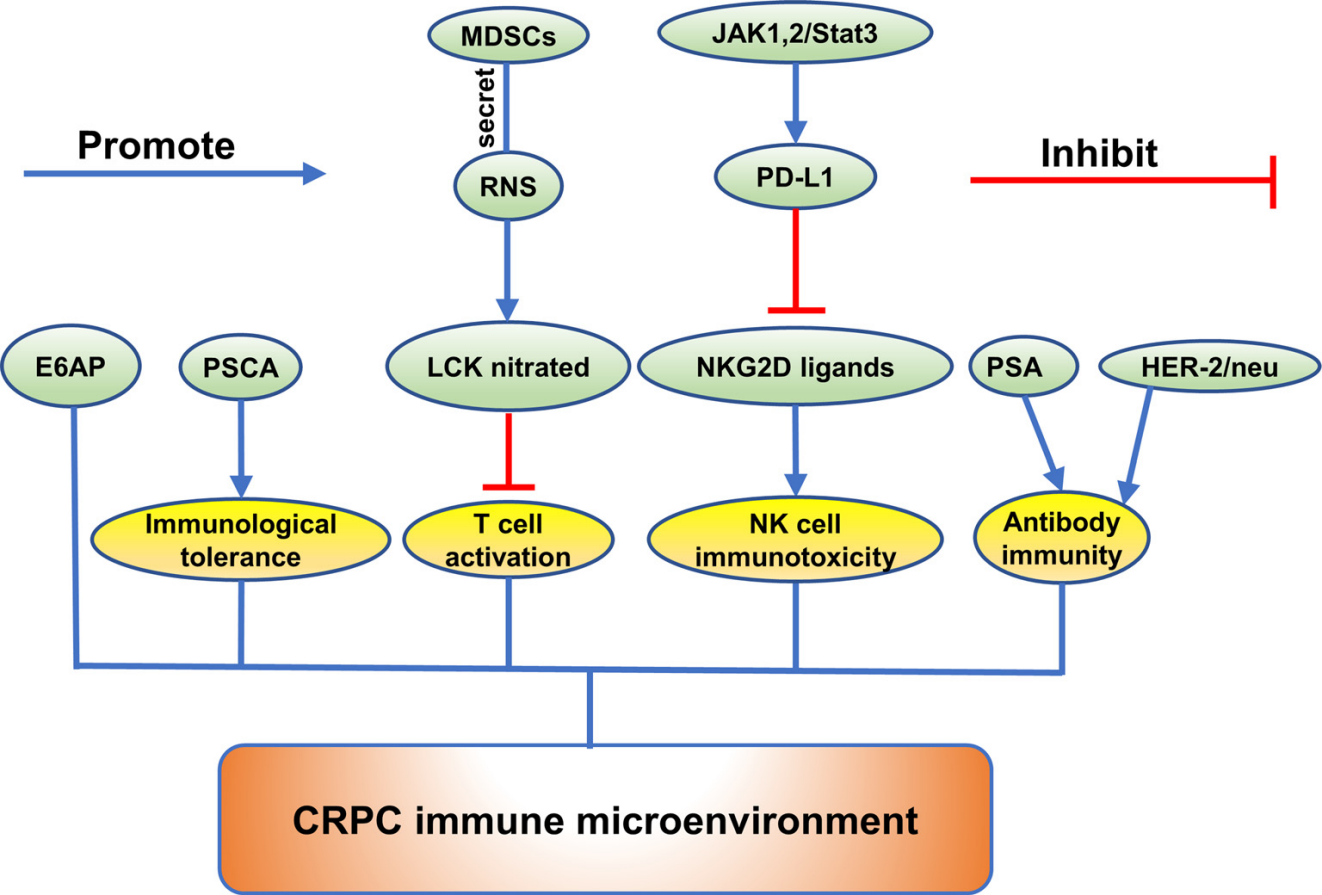
The role of emerging proteins in CRPC immune microenvironment.

Recently, a majority of studies were carried out to investigate the function of the emerging proteins. It was widely verified that proteins could play a crucial role in growth, invasion, and metastasis of CRPC. (Exhibited in **Figures 1**–**3**).

### CRPC Growth

Many proteins have been shown to play a key role in the growth of CRPC through gene silencing and other gene editing methods. Knockdown of RNF6 was found to attenuate the growth of CWR-R1 and C4-2B in androgen-depleted media (13). Additionally, it was found that RNF6 may function by regulating AR target genes and suppressing androgen-suppressed gene BMF which was a proapoptotic member of BCL2 family proteins (13). In addition, silencing of SEMA3C resulted in reduced cell growth that was rescued by the addition of recombinant SEMA3C: Fc in DU145 cells (11). It was discovered that increased SEMA3C expression was correlated with CRPC bone metastases (11). Yu Ishizuya et al. discovered that ACTN4 was increased in DU145 cells as well as exosomes from this cell line (8). Downregulation of ACTN4 expression by siRNA in DU145 cells significantly attenuated cell growth (8). Checkpoint Kinase 2 (CHK2) were identified to play a role in CRPC growth (73). To identify signaling pathways regulating cell growth of PCa, a high-throughput RNAi screen was used by Huy Q Ta et al. and discovered that knockdown of CHK2 significantly increased the growth of PCa and hypersensitized cells to low androgen levels (73). Similarly, knockdown of epithelial cell adhesion molecule (EpCAM) resulted in decreased proliferative and clonogenic ability in PC-3, DU145, and C4-2B cells (32). Another study demonstrated that after transfecting LNCaP-AI cells with p66Shc shRNA, there was a reduced cell proliferation in LNCaP-AI cells (28).

In contrast, overexpression of FUT8 in prostate cancer cells was discovered to promote the proliferation of cells in androgen-ablated conditions (35). In addition, FUT8 overexpression by transfecting plasmid DNA carrying FUT8 gene contributed to increased level of cell surface epidermal growth factor receptor (EGFR) and corresponding downstream signaling, resulting in increased cell survival in androgen-depleted conditions (7).

The Rab-proteins and endosomal sorting complexes crucial for transport (ESCRT) machinery were important in vesicular trafficking and secretion (74, 75). RAB27A, RAB27B, and VPS36, which were components of rab-proteins and ESCRT machinery, respectively, were verified to be prognostic biomarker for patients with localized PCa (76). It was shown by qRT-PCR that PCa tissue expressed low levels of VPS36, especially in CRPC tissues (76). Interestingly, knockdown of RAB27B and VPS36 significantly decreased colony formation but did not reduce the growth of PC3 cells (76). Golgi phosphoprotein 3 (GOLPH3) has been identified to be a highly conserved Golgi membrane protein using proteomic analysis of the Golgi apparatus and showed to regulate the mitochondrial lipids as a mitochondrial protein (77–79). GOLPH3 protein expression was significantly increased in PCa, especially in two androgen-independent cell lines, PC-3 and DU145 (80). Silencing GOLPH3 expression suppressed cell proliferation of PC-3 by inhibiting phosphorylation of AKT and mTOR (80). In addition, aspartyl (asparaginyl) βhydrolase (ASPH) is a transmembrane protein and it was reported that ASPH silencing decreased cell proliferation, invasion, and cyclin D1 expression level through regulation of the NOTCH signaling (39). Similarly, Masahiro Sugiura et al. found that AR-V7 knockdown lead to suppression of CRPC growth by activating both common AR/AR-V7 target and specific AR-V7 target (15).

Moreover, transcriptional repressor REST was found to play a role in CRPC cell proliferation (5). REST depletion resulted in cell cycle arrest in G1, which could be rescued by knockdown of p53 (5). Therefore, it was indicated that REST loss had a negative regulation of cell cycle progression by the activation of the p53 pathway (5). Similarly, Arnaud Blomme et al. found that DECR1 deletion led to decreased CRPC tumor growth (6). The normal epithelial cell-specific-1 (NES1) gene is also named as KLK10 which is a member of the kallikrein family and encodes human kallikrein 10 (hK10) (81). It was reported that KLK10 decelerated CRPC proliferation (82). Jiajia Hu et al. obtained a high-purity KLK10-expressed stable cell line PC3-KLK10 by reconstructed lentiviral vector, and they found that over-expressing KLK10 in PC3 could decrease tumor proliferation and increase apoptosis and inhibit glucose metabolism at the same time (82). Furthermore, a negative feedback loop was investigated between KLK10 and Bcl-2/HK-2 (82).

Some studies used drugs to inhibit protein function to study its effect on the growth of CRPC. YAP1 and PAK2 were also reported to regulate the growth in CRPC (19). A MS-based quantitative proteomic approach was implemented and YAP1 and PAK2 were identified to change in phosphorylation comparing protein phosphorylation in orthotopic xenograft tumors which were grown in either intact or castrated mice (19). Furthermore, elevated levels of PAK2 and YAP1 in clinical samples of CRPC were also demonstrated (19). PAK2 regulated cell proliferation and mitotic timing (19). The growth of androgen-independent PC3 xenografts could be inhibited by pharmacologic inhibitors of PAK2 (PF-3758309) and YAP1 (Verteporfin) (19). Similarly, Oleg Tatarov et al. found that src inhibition by dasatinib resulted in reduced migration and proliferation of CRPC cells (10).

In addition, Serum and glucocorticoid-induced protein kinase 1 (SGK1), a member of the ‘AGC’ subfamily, was reported by us to play an important role in cancer development (83). We have previously investigated that inhibition of SGK1 by either GSK650394 or SGK1 shRNA led to decreased cell viability in PC3 cells by inducing autophagy-dependent apoptosis *via* the mTOR-Foxo3a pathway (84).

Signaling axis also has been found to promote the growth of the CRPC. Matrix metalloproteinases 9 (MMP9) was found to correlate with AR protein expression in the tissues of primary and metastatic PCa and high MMP9 expression level correlated with poor prognosis (85). Constitutive activation of AR upregulated expression level of MMP9 and VEGF/VEGF receptors (85). Furthermore, it was shown that AR influences MMP9/VEGF signaling axis *via* PIP5K1α/AKT and MMP9 physically interacted with PIP5K1α through formation of protein–protein complexes (85). The elevated sequential activation of AR/PIP5K1α/AKT/MMP9/VEGF signaling axis resulted in increased invasiveness and growth of metastatic CRPC (85). Conversely, treated with PIP5K1α inhibitor, invasion activity of CRPC cells expressing activated AR were dramatically repressed (85). Similarly, Musashi2 (MSI2) belongs to the evolutionarily conserved Musashi RBP family and downregulation of MSI2 was reported to reduce CRPC cell proliferation by regulation of AR (86, 87). Moreover, IL-8 signaling was reported to regulate cyclin D1 expression and activate signal transduction pathways underpinning CRPC cell proliferation (88).

### CRPC Invasion and Metastasis

We have previously discovered that inhibition of SGK1 induced autophagy contributed to reduced EMT by the downregulation of snail, leading to decreased cell migratory ability of PC3 (89). Similarly, p66Shc was reported to promote the migratory activity of PCa cells including CRPC cells (27). Matthew A. Ingersoll et al. found that higher p66Shc expression was correlated with high cell migratory ability across several PCa cell lines (27). PCa cells expressing low levels of p66Shc could be induced to metastasize by peroxide treatment in a dose-dependent manner (27). Highly expressed p66Shc in PCa cells increased the cell migratory ability which could be attenuated by p66Shc shRNA transfection or expression of oxidase-deficient dominant-negative p66Shc W134F mutant (27). Furthermore, Rac1, a protein that was activated in p66Shc-elevated cells, was validated and it was shown that p66Shc promoted formation of lamellipodia through Rac1 activation indicating (27).These results indicated that p66Shc increases cell migratory ability through ROS-mediated activation of migration-associated proteins, especially Rac1 (27).

In addition, VCL and FLNC were also verified to play a critical role in CRPC cell metastasis (29, 30). Jianzhong Ai et al. discovered that knockdown of VCL and FLNC gene expression significantly decreased PCa cell metastasis (30). To analyze the link between chemokine CXCL1 and neutrophil-derived cytokines LCN2, Yongning Lu et al. found that CXCL1-LCN2 axis activates Src signaling and triggers the epithelial-mesenchymal transition (EMT), resulting in enhanced tumor metastasis (90).

Bromodomain and Extraterminal domain (BET) proteins (BRD2, BRD3, BRD4, and testis-specific BRDT) are a family of chromatin-associated proteins that could detect and bind to acetylated lysine residues on nucleosomal histone tails resulting in regulation of gene expression (91, 92). Jordan S. Shafran et al. discovered that BRD4 reduced cell migration of all models of CRPC, but BRD2 and BRD3 only modulated migration and invasion in less aggressive models which kept AR signaling (93). Moreover, these researchers found that BRD4 regulated CRPC cell migration and invasion through transcription of AHNAK (93).

ACTN4 was also identified to promote CRPC invasion (8). Knockdown of ACTN4 inhibited cell invasion in DU145 cells (8). In addition, knockdown of YAP1 and PAK2 both reduced cell colony formation and cell invasion activity also suggested an important factor in CRPC invasion (19). Similarly, AR-V7 positivity associated with a higher bone, or any site, metastasis in CRPC (16). Moreover, knockdown of EpCAM contributed to reduced invasion in PC-3, DU145, and C4-2B cells (32). Xiangchun Wang et al. found that loss of FUT8 in PC3 cells resulted in decreased cell motility (36). PSA was crucial in development of CRPC and Haoyong Li et al. investigated that PSA promoted the apoptosis *in vitro *(94). Conversely, PSA knockdown decreased tumorigenesis and metastasis of C4-2 cells *in vitro* and *in vivo (* 94). And PSA may promote the tumorigenesis by mediating MCM4 (94).

To identify the mechanism correlated with more distant metastasis, kinases differentially expressed between the CRPC cell lines PC-3 and PC-3M, which was a metastasis-derived variant of PC-3 cells, were analyzed by MS-based comparative phosphoproteome strategy (95). In this study, 151 phosphoproteins differently expressed between the CRPC cell lines PC-3 and PC-3M were identified (95). Seven motifs, -SP-, -SxxE-, -PxS-, -PxSP-, -SxxK-, -SPxK-, and -SxxxxxP-, were discovered to express a higher level in PC-3M cells compared to PC-3 cells (95). Based on these motifs, the kinases Ste20-like kinase (SLK), p21-activated kinase (PAK)2, mammalian Ste20-like kinase (MST)4, A-Raf proto-oncogene serine/threonine kinase (ARAF), and mitogenactivated kinase kinase (MAP2K)2 were increased in PC-3M cells relative to PC-3 cells, suggesting that MST4, MAP2K2, ARAF, PAK2, and SLK are kinases potentially associated with increased migratory ability (95).

### CRPC Metabolism

AR play a crucial role in regulation of metabolism in PCa cells throughout the transition from early-stage, androgen-sensitive CSPC to androgen-independent CRPC (96). It was reported that AR is reprogrammed to increase fatty acid synthesis (FAS) from glucose in CRPC cells (6). Comparing the proteomes of three pairs of CSPC and CRPC cell lines, the researchers found that most of the differentially expressed proteins between each pair function in metabolism (6). PPARG, PGC1A, NR1H2, NR1H3, GLUT1, ACC, and ACLY are all metabolism-related proteins and have changed during the development of CRPC (6). Knockdown of the AR reduced glucose utilization in CRPC cells and AR-depleted cells showed reduced expression level of ACC and ACLY (6). DECR1 was reported to regulate lipid homeostasis in CRPC (6). It was found that DECR1 involves in redox homeostasis in the way of maintaining a balance between saturated and unsaturated phospholipids (6). Knockout of DECR1 induced ER stress and made CRPC cells sensitive to ferroptosis (6). Moreover, DECR1 deletion decreased lipid metabolism of CRPC *in vivo (* 6). Another study discovered that knockdown of YAP1 led to restored lipid content and reduced free fatty acid level in enzalutamide-resistant cells (20).

In addition, it was demonstrated that sterol response element-binding protein (SREBP) family are transcriptional regulators that coordinately activate over 20 functionally related enzymes associated with lipid and cholesterol synthesis (97). Susan L. Ettinger et al. found SREBPs and their downstream effector genes increased during progression to CRPC in the LNCaP xenograft model of PCa and contributed significantly to cell metabolism (97). Insulin-Like Growth Factor (IGF) family were shown to increase uptake of glucose and protein synthesis, whereas reducing serum free fatty acids (FFA) and hepatic glucose production (98). In addition, this family plays a key role in androgen-independent progression (98).

Interestingly, a study reported that the mitochondrial oxidation phosphorylation (mOxPhos) significantly decreased in castration-resistant LNCaP-95 cells relative to LNCaP cells (37). Comparing the wild type with androgen ablated LNCaP-95 cells, a lot of proteins in the OxPhos complexes reduced in the LNCaP-95 castrated resistant cells (37). Complex I, the major entry point for electrons to the respiratory chain and the rate-limiting step in overall respiration, was discovered to have many protein subunits downregulated, indicating the dysfunction of NADH dehydrogenase (37). A similar lower expression of cytochrome c oxidase, cytochrome c reductase in complex IV and the ATPase in complex V, were also investigated in the LNCaP-95 androgen resistant prostate cancer cells (37).

Moreover, the aldo-keto reductase family 1 member C3(AKR1C3), the prostatic enzyme was found to increase in CRPC and confirmed to convert androstenedione (A) to testosterone (T) by real-time reverse transcription-PCR and immunohistochemistry (99). PKC could be activated with phorbol ester (TPA) (100). It was reported that the release of ethanolamine (Etn) was only specifically upregulated in the metastatic basal PC3 cell line on TPA treatment most probably by PKC activation of PLD1 and the turnover of ethanolamine phosphoglycerides (EtnPGs) was also increased (100). Moreover, it was found that Etn released from cells may decrease uptake of choline (Cho), influencing the membrane PtdEtn : PtdCho ratio and regulating the action of PtdEtn-binding proteins such as the anti-apoptotic hPEBP4 and RKIP (100). These results suggested that PKC regulated PLD1 and release of Etn then influenced uptake of Cho in PC3 cells (100).

### CRPC Immune Microenvironment

A study reported that reactive nitrogen species (RNS) secreted by myeloid-derived suppressor cells (MDSCs) could induce T cell tolerance (101). Shan Feng et al. discovered that lymphocyte-specific protein tyrosine kinase (LCK), an initiating tyrosine kinase in the T cell receptor signaling cascade and nitrated at Tyr394 by MDSCs, inhibited T cell activation and reduced interleukin 2 production (101). In the mouse model of CRPC, CRPC is resistant to an immune checkpoint blockade (ICB) therapy, while the results come out conversely when combined ICB with an RNS neutralizing agent, which indicated that RNS played an imported role in CRPC immunity (101).

In addition, programmed death−ligand 1 (PD−L1) was identified to be upregulated in CRPC cells induced by hypoxia, while addition of PD-L1 antibody increased expression of NKG2D ligand and the immune cytolytic activity of NK cells toward CRPC cells (102). It was discovered that inhibition of the Janus kinase (JAK)1,2/signal transducer and activator of transcription 3 (Stat3) signaling pathway reduced the PD−L1 expression in CRPC cells and increased expression of NKG2D ligands (102). In addition, inhibition of JAK1,2/Stat3 signaling pathway increased the sensitivity of CRPC cells to NK cell immunity (102). Christian D. Fankhauser et al. also found that PD-L1 was not expressed in benign prostatic hyperplasia (BPH) or localized PCa and was only expressed in a minority of CRPC tumors and infiltrating immune cells (103). Moreover, they discovered a moderate positive correlation between PanCK and PD-L1 expression (103).

Novel potential links of E6-Associated Protein (E6AP) with immune response have been exposed by pathway analyses in DU145 cells (104). Comparing the significantly changed transcripts and proteins following E6AP knockdown in DU145 cells, some transcripts were found to change consistently in the same direction as the proteins (104). Interferon signaling, interferon gamma signaling, and cytokine signaling in immune system were predominantly associated with these proteins (104). In addition, clusterin was identified as a novel target of E6AP (104). In contrast, overexpression of prostate stem cell antigen (PSCA) was found in both androgen-dependent and androgen-independent prostate cancers (105). PSCA is an autoantigen which can induce immunological tolerance and hardly incite effective immunologic response (105).

To find which prostate cancer associated proteins induce immune responses, Douglas G. McNeel et al. evaluated the inherent humoral immune response against p53 and HER-2/neu, PSA, prostatic acid phosphatase in 200 patients (106). The results showed that for PSA and HER-2/neu the prevalence of antibody immunity was increased in patients with androgen independent disease (106).

## Molecular Mechanism of Emerging Proteins in CSPC to CRPC

**Figure 4:**
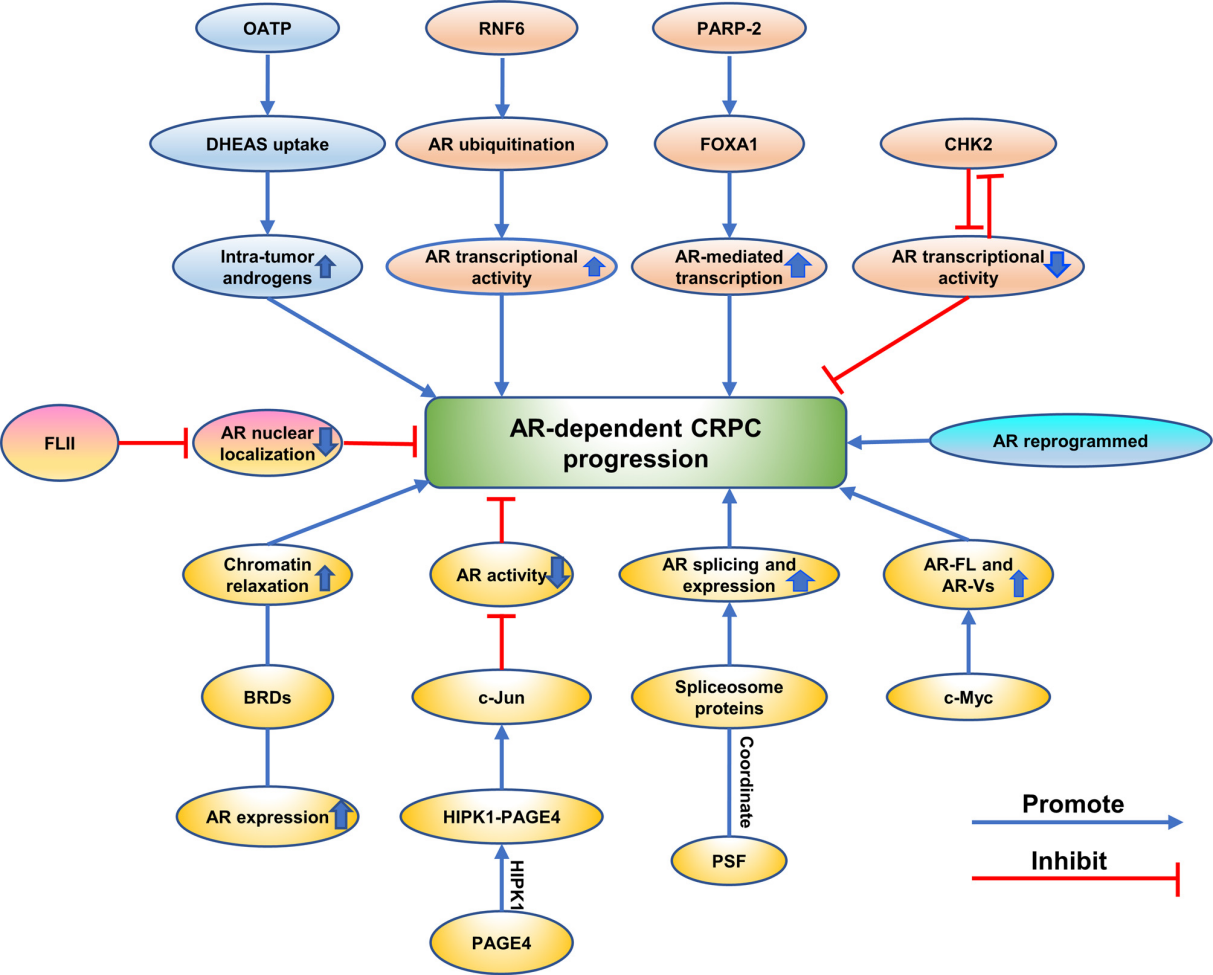
Emerging proteins mediate CRPC progression *via* AR-dependent way. The different mechanisms are showing as different colors.

**Figure 5:**
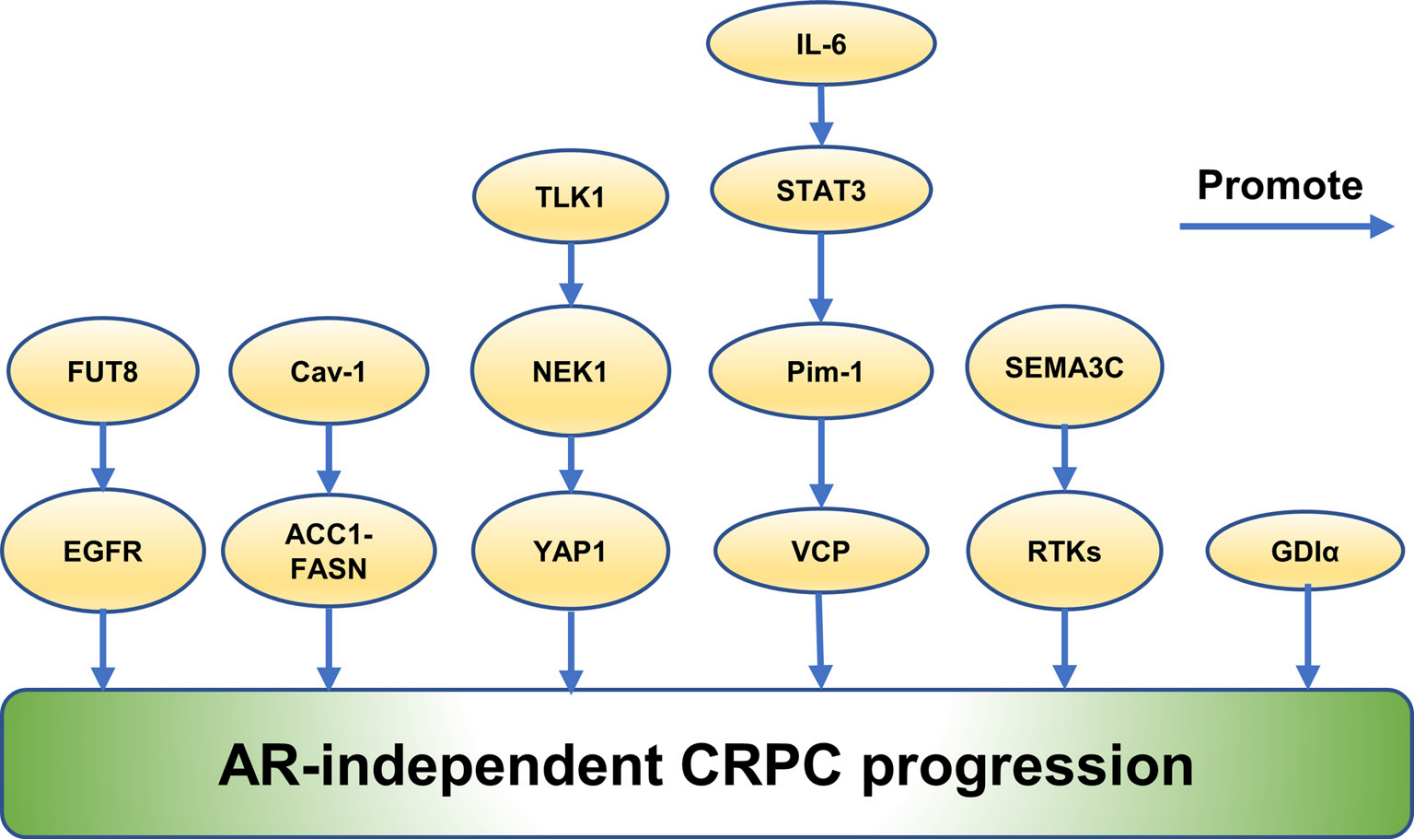
Emerging proteins mediate CRPC progression *via* AR-independent way.

The mechanisms of prostate cancer progression to CRPC associated with proteins are complex and mainly involve the AR-dependent and AR-independent mechanisms. (Summarized in **Figures 4** and **5**).

### Emerging Proteins Mediate CRPC Progression *via* AR-Dependent Way

Some proteins promote CRPC progression by regulating intra-tumoral levels of androgens in CRPC. Epidemiologic and *in vitro* studies demonstrate OATP transporters exert an effect on the response of PCa to therapy of androgen deprivation by regulating intra-tumor androgens (107). It also has previously been shown that castration resistant metastases express higher levels of multiple SLCO transporters than primary PCa (108). Based on these studies, Sean Green, PhD et al. examined uptake of androgen in LNCaP cells *in vivo* which were engineered to express OATP transporters and found that LNCaP cells expressing OATP1B1 and 2B1 showed an increased uptake of DHEAS resulting in a higher level of intra-tumor androgens, suggesting that OATP transporters promote CRPC progression in an AR-dependent way (107). Proteins contributed to CRPC progression by changing the localization of AR were also verified. Flightless I (FLII) is a member of the gelsolin superfamily of actin-remodeling proteins and functions as a transcriptional coregulator (109). It was reported to inhibit CRPC progression through targeting androgen receptor signaling (109). A positive correlation between the expression of FLII and overall survival of prostate cancer patients expressing high levels of AR expression was shown by a clinical gene expression array dataset (109). The protein and mRNA levels of FLII decreased in human prostate cancers (109). FLII binds with AR through the ligand-binding domain of AR and a competitive binding to AR was observed between FLII and the ligand (109). These researchers found that FLII inhibited AR transactivation and reduced AR nuclear localization (109). FLII contributed to growth of prostate cancer cells through AR-dependent signaling, and reintroducing FLII in CRPC cells sensitized the cells to endocrine therapies (109).

Other proteins regulated CRPC progression by controlling AR splicing and expression. Global changes in chromatin accessibility were known to promote cancer progression in the way of reprogramming transcription factor binding which was associated with histone acetylation readers such as BRD4 (110). Chromatin accessibility was verified to define CRPC. It was discovered that deregulation of AR expression drives chromatin relaxation mediated by AR/androgen-regulated bromodomain-containing proteins (BRDs) (110). Furthermore, the author reported that BRDs were highly expressed in CRPCS and that ATAD2 and BRD2 had prognostic value (110). Consistent with prior reports, Jonathan Welti et al. found that nuclear BRD4 protein expressed higher level in CRPC than treatment-naive biopsies in the same patient and higher expression at diagnosis correlated with worse outcome (111). The expression of BRD2, BRD3, and BRD4 RNA in CRPC biopsies associated with AR-driven transcription (111). AR-V7 expression and AR signaling could be decreased by Chemical BETi and knockdown of combined BET family protein (111). BETi reduced persistent AR signaling and growth of a xenograft model derived from CRPC patient with AR amplification and AR-V7 expression (111). RNA-binding protein PSF was also shown to contribute to CRPC progression by regulating splicing activities for AR expression (112). PSF targeted at mRNAs of spliceosome-related genes and promoted AR splicing and expression by coordinating these spliceosome proteins to form a complex (112). Moreover, high-speed sequence analysis was used and found that PSF stabilized and activated key long noncoding RNAs and AR-regulated gene expressions in prostate cancer cells (112). Activation of AR and the expression of its variants along with the downstream signals are crucial for CRPC progression (112). It was known that overexpression of the full-length androgen receptor (AR-FL) and AR splice variants (AR-Vs) promote the progression of CRPC (113). A striking positive correlation between the level of c-Myc and the activity of the AR pathway, between the activity of the c-Myc pathway and the level of individual AR isoforms, and between the activities of the two pathways, was discovered (113). Furthermore, the c-Myc signature is highly expressed in tumors with high levels of AR, as is the AR signature in c-Myc-high-expressing tumors (113). c-Myc regulation of activity and expression of AR-FL and AR-Vs was confirmed in a patient-derived xenograft model and cell models, suggesting that c-Myc promoted CRPC through the regulation of activity and expression of AR-FL and AR-Vs (113). In addition, Prakash Kulkarn et al. discovered that when PAGE4 is phosphorylated by HIPK1, HIPK1-PAGE4 enhanced c-Jun and suppressed AR activity in CRPC cells (58).

There are lots of studies showing that proteins changed AR transcriptional activity to mediate CRPC progression. An ubiquitin E3 ligase RNF6 was identified as one of AR-associated proteins by proteomic screen and may contribute to CRPC progression (13). RNF6 was found to induce AR ubiquitination and increase AR transcriptional activity (13). Mutation of RNF6-induced ubiquitination acceptor sites on AR or specific knockdown of RNF6 selectively regulates expression levels of a subset of AR target genes and attenuates recruitment of AR and its coactivators to androgen-responsive elements which were present in the regulatory region of these genes (13). In addition, RNF6 is overexpressed in human CRPC tissues and critical for growth of prostate cancer cells under androgen-depleted conditions, suggesting that RNF6-induced ubiquitination may alter AR transcriptional activity and specificity by modulating co-factor recruitment and played a role in CRPC progression (13). Another study reported that knockdown of CHK2 hypersensitized PCa cells to low androgen levels indicating a crucial role of CHK2 in progression to CRPC (73). The study found that CHK2 exerted its effects dependent on CDC25C and CDK1 which were downstream signaling proteins (73). In addition, CHK2 depletion promoted androgen receptor (AR) transcriptional activity on androgen-regulated genes, validating the finding that CHK2 influences PCa proliferation through the AR partly (73). Interestingly, the author further validated that CHK2 was a novel AR-repressed gene, indicating that there existed a negative feedback loop between AR and CHK2 (73). In addition, CHK2 physical association between CHK2 and AR was further discovered, and that cell cycle inhibition enhanced this association (73). It was also shown that CHK2 signaling was lost during prostate cancer transformation to CRPC (73). Moreover, Bin Guia et al. found that implementation of genetic or pharmacological means to target PARP-2 selectively blocks interaction between FOXA1 and PARP-2, which in turn decreased AR-mediated transcription and inhibited anchorage-independent growth of LNCaP cells and AR-positive CRPC cell growth (9). It can be inferred that PARP-2 have an influence on development of CRPC through transcriptionally modulating AR-mediated gene expression (9).

In a recent report, Arnaud Blomme et al. found that active AR signaling and nuclear AR were observed in CRPC cells and AR was reprogrammed to promote CRPC progression (6). Acquired metabolic phenotype was common in CRPC cells and correlated with perturbed glucose and lipid metabolism (6). Knockdown of the AR impaired glucose utilization and reduced expression of ACC and ACLY in resistant cells, suggesting that AR is reprogrammed to promote fatty acid synthesis from glucose in resistant cells and promote CRPC progression (6). DECR1 increased in ARI-resistant cells compared to WT LNCaP and maintains lipid homeostasis in CRPC cells (6). Further study revealed that the expression of DECR1 was required for growth of CRPC tumor *in vivo *(6).

Hence, these findings augment investigators’ comprehension of CRPC progressions *via* the consideration of proteins and AR-dependent signaling pathways.

### Emerging Proteins Mediate CRPC Progression *via* AR-Independent Way

FUT8 was identified to increase in CRPC indicating a role in CRPC progression (7). Naseruddin Höti et al. found that overexpression of FUT8 contributed to increase the level of cell surface epidermal growth factor receptor (EGFR), and its relevant downstream signaling, resulting in increased cell survival of PCa in androgen-depleted conditions (7). The coregulatory mechanisms of FUT8 and EGFR expression in CRPC xenograft models were studied furthermore and it was shown that castration induced FUT8 overexpression correlated with elevated expression of EGFR (7). The findings suggested that FUT8 played an important role in escaping castration-induced cell death and promoting the progression of CRPC (7).

Similarly, it was reported that Caveolin-1 (Cav-1) regulates castration resistance through ACC1-FASN upregulation in an AR-independent way and lipid synthesis (114). Theodoros Karantanos et al. discovered that overexpression of Cav-1 promoted castration resistance in PTENcKO tumors (114). Cav-1 altered the expression of ACC1 and FASN in PCa cells in an AR-independent way at the transcriptional level and promoted synthesis of palmitate (114). Inhibition of FASN was more effective in Cav-1-overexpressing cells (114). Cav-1 induction increased the expression of ACC1 and FASN in PTENcKO tissues and reduced the apoptotic effects of castration, which increased Cav-1 and ACC1 expression (114). Furthermore, the researchers found that FASN was a crucial actor in the survival of PCa cells which expressed Cav-1 under androgen deprivation (114). Upregulation of palmitoleate and oleate correlated with a poor response to AA in the bone marrow aspirates from mCRPC (114).

Interestingly, a study reported that RhoGDI (GDI) α decreased in LNCaP-IL6+ cells, which was generated by treating LNCaP cells chronically with interleukin-6 (IL-6) and grew in a hormone resistant way, comparing with LNCaP cells (115). GDIα overexpression inhibited PCa cells growth and sensitized LNCaP-IL6+ cells to androgen deprivation (115). Conversely, GDIα downregulation promoted androgen-sensitive LNCaP cells growth in androgen-deprived state, suggesting that GDIα promoted development of CRPC in an AR-independent way (115).

It was known that IL-6 played a role in the development of CRPC (116). IL-6 expression are increased in CRPC patients and regulate AR activity (116). Proteomic analysis revealed expression of 27 proteins changed in LNCaP cells compared with cells without IL-6 treatment and valosin-containing protein (VCP)/p97 plays a crucial role in co-regulation of altered proteins (116). Moreover, it was discovered that IL-6 induced VCP expression through Pim-1 *via* STAT3 activation is AR independent, suggesting that VCP played a role in CRPC (116).

In addition, activation of growth factor receptor tyrosine kinase (RTK) pathway is a crucial mechanism in mediating cancer survival, growth and treatment resistance (11). James W Peacock discovered that SEMA3C drives activation of multiple RTKs including EGFR, ErbB2, and MET in a cognate ligand-independent manner *via* Plexin B1 (11). SEMA3C increased in CRPC, where it promoted growth and castration resistance of cells. Inhibition of SEMA3C delays progression of CRPC and enzalutamide-resistance (11). These results indicate that SEMA3C promoted CRPC through RTK signaling, which was also aindependent of AR (11).

A recent report discovered that TLK1>NEK1>YAP1 axis plays an important role during the progression to CRPC (117). Md Imtiaz Khalil et al. previously demonstrated that TLK1>NEK1>ATR>Chk1 axis is a crucial actor in cell cycle arrest when CSPC cells suffered androgen deprivation (117). The overexpression of wt-NEK1and YAP1 contributed to a rapid transition to growth of CRPC (117). Higher expression of wt-NEK1 associated with accumulation of YAP1, indicating that TLK1>NEK1>YAP1 axis led to adaptation to CRPC growth (117). NEK1 could be co-immunoprecipitated with YAP1 (117). YAP1 was found to be phosphorylated by NEK1 on six esidues *in vitro*, which might enhance its interaction with transcriptional partners and promote reprogramming of the cells toward CRPC (117).

## The Clinical Application of Proteins in CRPC

The methods commonly used for CRPC screening include a rise in PSA, bone scan, biopsy, and/or PET imaging of recurrent/new metastases (3). But diagnostic accuracy is low and CRPC progression is not monitored in the best way.

### Proteins as a Diagnostic Marker

Sharp et al. explored nuclear AR-V7 expression in same-patient, matched biopsies, as 63 patients progressed from CSPC to CRPC. They found that nuclear AR-V7 protein significantly increased from CSPC to CRPC (17). Nuclear AR-V7 can be detected by immunohistochemistry and could be a good diagnostic marker for CRPC. Serum B2M levels were increased in metastatic CRPC patients, and B2M was more specific for androgen stimulation under the conditions tested compared with PSA (66). What’s more, B2M can be easily detected in serum, which increases potential applicability to guided therapy of CRPC. In addition, enzymes of the ketogenic pathway are increased in an androgen-independent derivative of LNCaP cell line and LuCaP 96AI castration-resistant xenograft (33). These enzymes might be potential biomarkers for CRPC diagnosis but further studies are needed to confirm this (33).

### Proteins as a Prognostic Marker

Recently, research has found that some proteins might be potential biomarkers for CRPC prognosis, such as SRC and FLII (10, 109). Increased SFK activity was associated with reduced time from biochemical relapse to death (10). Furthermore, increased SFK activity in AIPC patients may have higher transfer potential (10). Similarly, higher AR-V7 in CRPC biopsies was associated with shorter PSA-PFS (118). In addition, CRPC-specific AR-V7 protein expression and positive protein detection were significantly associated with worse outcomes (17). In conclusion, AR-V7 has been reliably detected in many types of human biological samples and thus can serve as a negative prognostic biomarker. High DECR1 expression was found to associate with poor prognosis in CRPC (6). Moreover, DECR1 overexpression dramatically decreased patient disease-free survival and was associated with worse outcome in metastatic patients (6). Additionally, other proteins with prognostic potential such as ASPH, BDH1, HMGCL, HMGCS2, ACAT1, OXCT1, etc. were also discovered in different research (33, 34, 39). These proteins are all potential prognostic markers and can be detected by immunohistochemistry. In the future, they can be used in combination to stratify CRPC risk, so as to give patients more personalized and reasonable treatment.

### Proteins as a Predictive Marker

Although progress has been made in the therapy for CRPC in recent years, an urgent challenge still exists because there are fewer predictive biomarkers for CRPC compared to prognostic biomarkers (119). Therefore, implementation of proteins as biomarkers has great potential. In a study, Eleni Efstathiou et al. found that the absence of AR-V7 in bone marrow biopsies from mCRPC correlated with better treatment response to enzalutamide (120). CRPC patients with AR-V7 positive CTCs were associated with resistance to enzalutamide and abiraterone (121, 122). This means that AR-V7 can be detected not only in tissues but also in CTCs, which are non-invasive and adds potential applicability for guided therapy with the development of AR-V7-assay. Similarly, inhibitor of de-ubiquitinase USP7 P5091 reduced levels of tumor suppressor protein CCDC6, sensitizing the CRPC cells to PARP inhibitors (123). Therefore, CCDC6 and USP7 can be potential biomarkers of sensitivity to PARP-inhibitors in CRPC (123). KENTA ONISHI et al. found that expression of KLG in primary prostate cancer lesions correlated with CRPC progression and KLG could be a predictor of resistance to docetaxel (124). Taken together, these proteins may be predictive biomarkers for CRPC and could also be detected by immunohistochemistry, which facilitates the selection of optimal treatment modalities for patients.

### Proteins as a Therapeutic Target

Good anti-tumor drugs can kill tumor cells specifically and effectively on the premise of not impairing healthy cells (125). However, many drugs used today to treat tumors have more or less side effects. The proteins that are regulated in CRPC tissue and influence progression will become promising candidates for therapeutic targets. Inhibition of SEMA3C by small molecules was discovered to have an effect on the growth of DU145 cell lines, which indicated SEMA3C could be a potential therapeutic target (12). Furthermore, SEMA3C is not required for homeostasis in adult tissues, so SEMA3C inhibition may be well tolerated in adults (11). Similarly, RORg antagonist targeting MDR1 expression resensitizes cross-resistant CRPC to taxanes (26). Both inhibitors are available and may be used clinically in the future. Other proteins may become therapeutic targets by using RNA interference technology. For example, downregulation of ACTN4 by RNA interference significantly reduced cell proliferation and invasion in DU145 cells (8). A 5α-steroid reductase ‘type 3 5α-steroid reductase’ (SRD5A3) was identified to overexpress in CRPC (126). Knockdown of SRD5A3 in prostate cancer cells contributed to a dominant decrease in DHT production and a significant reduction in cell viability, suggesting a role in maintenance of androgen–androgen receptor-pathway activity in CRPC cells and the enzymatic activity might be a potential target for therapy of CRPC (126). However, this strategy requires further study to develop an optimal delivery system to protect the stability of RNAi-based drugs and investigate indications of various diseases with these drugs in early and late clinical trials. There are some proteins requiring further study to clarify their potential as therapeutic targets. YAP1 and PAK2 were identified to regulate cell colony formation and cell invasion activity without obvious changes in the activity of AKT, MAPK, or mTORC1 pathways, indicating that PAK2 and YAP1 may be potential therapeutic targets of CRPC (19). Moreover, MAR-binding proteins that are regulated in the prostate cancer cells could improve our understanding of CRPC process, and these proteins were reported to be promising targets for prostate cancer therapy (127). In addition, Huy Q Ta et al. proposed that perturbing CHK2 signaling may be a novel therapeutic approach to sensitize CRPC to ADT and radiation (73). Other proteins with the potential of being therapeutic targets such as AR-V7, TBLR1, EpCAM, caspase 3, vimentin, catalase, ERG, etc. were also proposed in different studies (18, 22, 24, 31, 32).

Currently, AR-V7 is one of the most well-studied and most promising proteins used as a PCa biomarker. It may be used as a biomarker for diagnosis, prognosis, prediction, and treatment. Although the proteins we mentioned above play an important role in prostate cancer diagnosis, prognosis, and development, more research is still needed to better understand and use these proteins.

## Conclusions and Perspectives

The rapidly increasing number of studies have found that the expression of proteins changes and proteins play a crucial role in the development of CRPC. Summarizing the biological roles of these proteins in CRPC will help to find more markers for diagnosis, prognosis, prediction, and treatment. These potential markers provide new insight into the mechanism of CRPC development and can be combined with existing markers for better management of CRPC patients.

With the development of high-throughput proteomics technology, a large number of proteins have been found to play an important role in the tumorigenesis and development of prostate cancer. In this review, we summarize the changes of protein expression between CSPC and CRPC and the proteins that promote the growth, metastasis, and invasion of CRPC. Furthermore, we summarize the related mechanisms of proteins in promoting CRPC, mainly including AR-dependent and AR-independent mechanisms. Understanding the role of these proteins in the development of CRPC will help to develop drugs that target the corresponding proteins or signaling pathways. Inhibition of abnormally expressed proteins or signaling pathways can lead to more efficient treatment for patients.

Biopsies are invasive and are associated with complications such as bleeding and sepsis. In the future, numerous markers may be investigated in non-invasive body fluids. More studies will also focus on the other roles of these potential markers in CRPC, as well as in the clinical application. In addition to proteins, other types of markers will also be further evaluated, such as IncRNA and exosomes. Moreover, further development of proteomic technologies will also promote the understanding of markers.

## Author Contributions

PK and LZ finished the manuscript and abstract; ZZ and KF consulted relevant literatures and completed English revision; YS, XD, CL, and TS completed the figures and tables; WL and ZT provided constructive feedback and guidance; WL completed critical revisions and proofread the manuscript. All authors have read and approved the final manuscript.

## Funding

This study was supported by grant from the National Natural Science Foundation of China Youth Science Foundation Project (Grant nos. 81802571), and Zhejiang Medical and Health Science and Technology Project (2019RC039).

## Conflict of Interest

The authors declare that the research was conducted in the absence of any commercial or financial relationships that could be construed as a potential conflict of interest.

## Publisher’s Note

All claims expressed in this article are solely those of the authors and do not necessarily represent those of their affiliated organizations, or those of the publisher, the editors and the reviewers. Any product that may be evaluated in this article, or claim that may be made by its manufacturer, is not guaranteed or endorsed by the publisher.

## Glossary

**Table:**

ADT	androgen deprivation therapy
AR	androgen receptor
ACTN4	actinin-4
AP-1	Activator Protein-1
ARI	AR inhibitors
ASPH	aspartyl (asparaginyl) β hydrolase
ARAF	A-Raf proto-oncogene serine/threonine kinase
AKR1C3	aldo-keto reductase family 1 member C3
AR-FL	full-length androgen receptor
AR-Vs	AR splice variants
A	androstenedione
B2M	β-2-microglobulin
BET	Bromodomain and Extraterminal domain
BPH	benign prostatic hyperplasia
BRDs	bromodomain-containing proteins
CSPC	castration-sensitive prostate cancer
CRPC	castration-resistant prostate cancer
CE3b	cryptic exon 3b
CLK2	CDC-Like Kinase 2
CLU	clusterin
CHK2	Checkpoint Kinase 2
Cav-1	Caveolin-1
Cho	choline
DECRI	2,4-dienoyl-CoA reductase
ETS	E26 transformation-specific
EpCAM	epithelial cell adhesion molecule
EMT	epithelial-mesenchymal transition
Etn	ethanolamine
EtnPGs	ethanolamine phosphoglycerides
E6AP	E6-Associated Protein
EGFR	epidermal growth factor receptor
FUT8	alpha (1, 6) fucosyltransferase 8
FLNC	filamin C
FLII	Flightless I
FAS	fatty acid synthesis
FFA	free fatty acids
GO	gene ontology
GOLPH3	Golgi phosphoprotein 3
GDI	RhoGDI
HIPK1	Homeodomain-Interacting Protein Kinase 1
IGF	Insulin-Like Growth Factor
ICB	immune checkpoint blockade
IL-6	interleukin-6
JAK	Janus kinase
LC	Liqui Chromatography
LCK	lymphocyte-specific protein tyrosine kinase
MDR1	multidrug resistance 1
MS	Mass Spectrometer
MMP9	matrix metalloproteinases 9
MSI2	Musashi2
MST	mammalian Ste20-like kinase
MAP2K	mitogenactivated kinase kinase
MDSCs	myeloid-derived suppressor cells
mOxPhos	mitochondrial oxidation phosphorylation
NES1	normal epithelial cell-specific-1
PSA	prostate-specific-antigen
PET	positron emission tomography
PCa	prostate cancer
PARP	Poly (ADPribose) Polymerase
PAGE4	Prostate-Associated Gene 4
PAK	p21-activated kinase
PDL1	programmed deathligand 1
PSCA	prostate stem cell antigen
RNF6	Ring finger protein 6
RNS	reactive nitrogen species
RTK	receptor tyrosine kinase
SFKs	Src family kinases
SEMA3C	Semaphorin 3C
SLK	Ste20-like kinase
SREBP	sterol response element-binding protein
Stat3	signal transducer and activator of transcription 3
SGK1	serum and glucocorticoid-induced protein kinase 1
TMAs	tissue microarrays
TMPRSS2	transmembrane serine protease 2
TBLR1	transducin beta like related 1
TIMP-1	tissue inhibitor of metalloproteinase 1
TPA	phorbol ester
T	testosterone
VCL	vinculin
VCP	valosin-containing protein
WB	western blotting
YAP1	yes-associated protein 1
